# The Use of Hybrid CNN-RNN Deep Learning Models to Discriminate Tumor Tissue in Dynamic Breast Thermography

**DOI:** 10.3390/jimaging10120329

**Published:** 2024-12-21

**Authors:** Andrés Munguía-Siu, Irene Vergara, Juan Horacio Espinoza-Rodríguez

**Affiliations:** 1 Department of Computing, Electronics and Mechatronics, Universidad de las Américas Puebla, Sta. Catarina Martir, San Andrés Cholula 72810, Mexico; andres.munguiasu@udlap.mx; 2 Department of Immunology, Instituto de Investigaciones Biomédicas, Universidad Nacional Autónoma de México, Mexico City 04510, Mexico; irene.vergara@iibiomedicas.unam.mx

**Keywords:** breast cancer, deep learning, neural networks, CNN-RNN, thermography, binary classification

## Abstract

Breast cancer is one of the leading causes of death for women worldwide, and early detection can help reduce the death rate. Infrared thermography has gained popularity as a non-invasive and rapid method for detecting this pathology and can be further enhanced by applying neural networks to extract spatial and even temporal data derived from breast thermographic images if they are acquired sequentially. In this study, we evaluated hybrid convolutional-recurrent neural network (CNN-RNN) models based on five state-of-the-art pre-trained CNN architectures coupled with three RNNs to discern tumor abnormalities in dynamic breast thermographic images. The hybrid architecture that achieved the best performance for detecting breast cancer was VGG16-LSTM, which showed accuracy (ACC), sensitivity (SENS), and specificity (SPEC) of 95.72%, 92.76%, and 98.68%, respectively, with a CPU runtime of 3.9 s. However, the hybrid architecture that showed the fastest CPU runtime was AlexNet-RNN with 0.61 s, although with lower performance (ACC: 80.59%, SENS: 68.52%, SPEC: 92.76%), but still superior to AlexNet (ACC: 69.41%, SENS: 52.63%, SPEC: 86.18%) with 0.44 s. Our findings show that hybrid CNN-RNN models outperform stand-alone CNN models, indicating that temporal data recovery from dynamic breast thermographs is possible without significantly compromising classifier runtime.

## 1. Introduction

Breast cancer (BC) is the most diagnosed malignancy and the second leading cause of death due to malignant tumors worldwide [1]. Early detection plays an essential role in decreasing the death rate caused by this type of cancer [2,3]. The gold standard for breast cancer diagnosis is mammography. This technique, however, has low sensitivity in dense tissue and exposes the patient to ionizing radiation [4,5,6]. The FDA approved thermography as a complementary imaging technique to mammography for the detection of breast cancer. However, the sensitivity was low and hardly differentiated between healthy and diseased tissue [7]. Infrared thermography is a non-invasive, rapid, and low-cost imaging technique [8]. The principle of this technique is the absorption of infrared (IR) light (7.5–13 µm) emitted by the surface of a body (e.g., breast) to generate a heat map and obtain its spatial temperature [9]. There are two acquisition protocols for infrared thermography: static infrared thermography (SIT), which registers corporal patterns in basal temperature, and dynamic infrared thermography (DIT), which studies the vascular physiological response after applying a thermal stimulus [10]. Breast cancer diagnosis in infrared imaging results in higher accuracy when using DIT, increasing the metric from 54% to 82% due to the enhancement of the vascular pattern after the thermal stimulus is removed [10,11,12].

Human interpretation and analysis of medical images is a time-consuming and error-prone task, so machine learning (ML) models have been widely used in breast cancer diagnosis [13,14,15,16,17,18]. A limitation of ML models such as support vector machines, decision trees, or naive Bayes is that they require manual intervention [19,20,21]. Deep learning techniques have been used in medical imaging since they can automatically identify complex patterns or extract the most relevant features from breast cancer images [5,13,20,21,22,23]. Deep neural networks (DNNs) have become particularly promising for disease classification, especially convolutional neural networks (CNNs) in diagnostic medical images [24,25]. CNNs are based on vision perception from significant inputs such as images; they extract visual features automatically through convolutional layers and classify them based on the training data [26]. These networks help diagnose the state of patients with various diseases, such as diabetes [27], cardiomegaly [28], coronavirus disease 2019 (COVID-19) [29], and breast cancer [30], using thermographic images. Another type of DNN known as a recurrent neural network (RNN) links their nodes to process information sequentially, making it more efficient for time-series data [31]. Recently, RNNs have been used in the medical field for breast cancer imaging classification [32,33] due to their ability to extract dynamic patterns using sequential data. In fact, there is evidence that hybrid CNN-RNN deep learning models are more effective than single deep learning models such as CNNs or recurrent neural networks (RNNs) in breast cancer detection modalities such as histopathology images [34,35,36], mammography [32], and ultrasound [37].

Deep learning models have made significant advancements in the segmentation and classification of medical images, leading to improved accuracy in critical clinical applications. According to Zhao et al. [38], the RGGC-UNet deep learning framework can be effective for semantic segmentation of signet ring cells (SRCs) in pathological images. This model improves segmentation accuracy while reducing computation complexity by incorporating residual ghost blocks and ghost coordinate attention. Aside from that, Salehi et al. [39] provide an in-depth analysis of CNNs and transfer learning in medical imaging. The study highlights the critical role of medical imaging in disease diagnosis and treatment, focusing on how CNN-based models can be used to improve image analysis and classification. It emphasizes the benefits of transfer learning, particularly for small datasets and limited computational resources, as well as improving accuracy, reducing time and resource requirements, and addressing class imbalances. Various CNN architectures are discussed in Mohammed et al. [40], emphasizing their effectiveness in automating medical image processing tasks, which are traditionally time-consuming, error-prone, and costly. It focuses on advancements in CNN design, such as hybrid models that combine CNNs with transformers and other architectures to improve the classification and segmentation performance of medical images.

Different techniques have been used in existing methods for segmentation, feature extraction, or training of a supervised ML model for breast cancer classification in thermal imaging, but many of the models used do not consider the temporal component of the dynamic acquisition protocol. Therefore, the lack of an automatic system that analyzes the sequence of images obtained by the DIT protocol and detects breast cancer disease motivated this research. Our study provides a novel framework that employs a hybrid CNN-RNN architecture for automatic breast cancer classification on images acquired using dynamic infrared thermography. CNNs are used to extract detailed spatial features from thermographic images, capturing localized temperature patterns and structural anomalies. Meanwhile, RNNs examine the temporal progression of these features throughout the image sequence. Consequently, this paper evaluates the performance (accuracy, sensitivity, and specificity) and efficiency (CPU execution time) of a deep learning model based on five state-of-the-art CNN architectures coupled with different types of RNN cells to determine whether tumor abnormalities are present or absent during dynamic breast thermography.

The contributions of this work are as follows:

- A hybrid deep learning model (CNN-RNN) to automatically extract spatial and temporal features from thermal breast images obtained by the DIT protocol to classify them into healthy or sick classes.
- A comparison in performance metrics of single and hybrid deep learning models for breast cancer diagnosis based on sequential thermographic images.
- Compute CPU execution time for single and coupled models using different neural networks to classify breast cancer under dynamic thermography.

## 2. Materials and Methods

### 2.1. Framework

**Figure 1:**
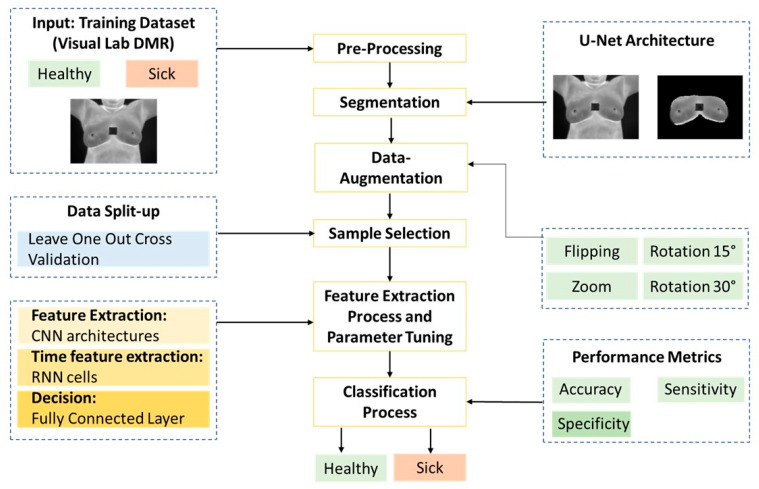
Diagram of the proposed methodology for binary breast cancer classification using hybrid CNN-RNN-based deep learning models.

The proposed binary classification system methodology for the breast thermal images obtained by the DIT acquisition protocol consists of the following steps: data cleansing and pre-processing, automatic segmentation, data augmentation, sample selection, and a coupled model (CNN-RNN) for classification and assessment with performance metrics for a balanced dataset as illustrated in Figure 1.

### 2.2. Dataset

The data were acquired from a public dataset from Antonio Pedro University Hospital known as Database for Mastology Research with Infrared Image (DMR-IR) [41] from 267 healthy volunteers and 44 sick volunteers. This database contains thermal breast images that were acquired using static and dynamic protocols. However, in the present work, dynamic images were used to extract the desired temporal features. Thermal images were obtained using the FLIR SC-620 IR camera. This camera possesses an image resolution of 640 × 480 pixels with an image frequency of 30 Hz; the spectral range detected by this camera is 7.5–13 µm, and the temperature range is from −40 °C to 500 °C. As part of the DIT protocol acquisition, a fan was used to apply a thermal stimulus to the volunteer until the thorax temperature reached an average of 30.5 °C. A sequence of frontal, each 15 s long, was then taken for 5 min. The data were stored in a txt file containing the spatial temperature in degrees Celsius of the heat map captured by the thermal camera.

### 2.3. Data Cleansing and Pre-Processing

**Figure 2:**
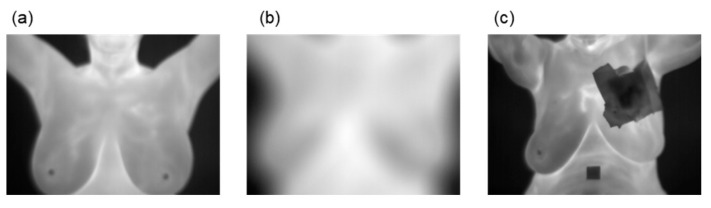
Sample grayscale thermograms from volunteers for a breast study: (**a**) The image is clear, so it is selected; (**b**) The image is blurry, so it is not selected; (**c**) The image contains material (bandaged breast) that covers the study region, so it is not selected.

Data cleaning consists of removing, replacing, or modifying data that may cause noise to the model to be trained [42]. We applied data cleaning to remove patients with less than 20 thermal images in sequence, as well as those with unclear images (noise or blurring) and those with unspecified material such as patches or implants over the central study region. Figure 2 shows an example of selected (a) and discarded (b and c) samples. After cleaning the data, we selected 166 labeled healthy samples and 38 diseased samples based on thermographic images suitable for the study. Finally, the number of volunteers labeled as healthy was matched with the number of sick volunteers to train the deep learning models with a balanced dataset, resulting in 38 volunteers for each class. The thermal images were resized to matrices of 224 × 224 to reduce the computational time of training tasks.

### 2.4. Segmentation

A fully automated segmentation was implemented using the U-Net architecture to remove noise from thermographic images such as necks, stomachs, and armpits in accordance with the methodology described by Mohamed et al. [22]. The U-Net architecture was employed to reduce the time required to segment each image manually for the use of editing software in all the thermal images. It has been reported that this network can be trained using a limited number of samples [43]. This network is convenient for biomedical data due to the large number of feature channels in its layers, which increases the resolution of the output [44]. We conducted a sample number sweep to train the U-Net to obtain the number of frames needed to segment the breast thermal images properly, starting the model training at 20 images with 5-frame increments. The appropriate number of samples to train U-Net for automatic segmentation of breast thermal images was 40. To segment the training images, we used the open-source software ITK-SNAP (version 3.8) for interactive image visualization and semi-automatic segmentation of medical images to crop only the region of interest (ROI), which, in this case, is the breasts.

#### U-Net Architecture

**Figure 3:**
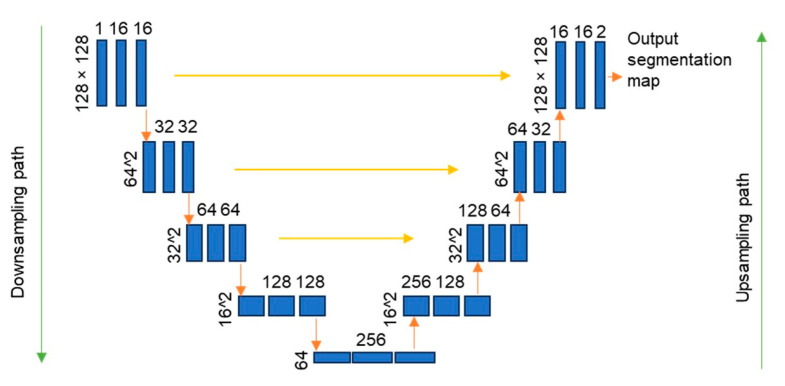
U-Net architecture. The contracting path is on the left side of the U-shape, and the expanding path is on the right. The blue boxes represent multi-channel feature maps. The number of channels is indicated on the top of the box. The x-y size is shown at the bottom left edge of the box. An orange arrow indicated each operation.

The U-Net is a fully connected layer (FCL) that automatically segments medical images [45]; it comprises 23 convolutional layers distributed in two network steps. The first part consists of the contracting path with a repeated convolution of 3 × 3, followed by a rectified linear unit (ReLU) and a max pooling for downsampling of the input. The next step is the expansive path; in this part, there is a concatenation in the feature maps of the contracting path to unsample the signal and crop the original image for the automated segmentation. The U-Net network is shown in Figure 3, where the convolutional layers are used to encode-decode the input data and crop the regions of the images in the trained network.

### 2.5. Data Augmentation

**Figure 4:**
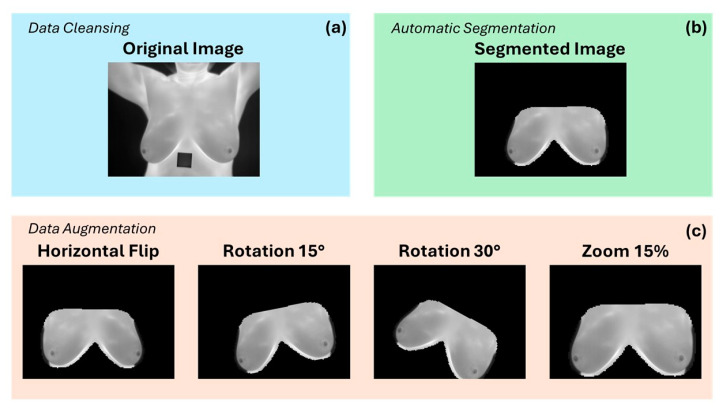
Example of a grayscale thermogram of the volunteer with ID 28: (**a**) selected image by data cleansing; (**b**) thermal image segmented using U-Net; (**c**) data augmentation using the transformations of horizontal flip, rotation 15°, rotation 30°, and zoom 15%.

Once the thermal images were cropped to the ROIs, the data augmentation was applied using four different transformations: a horizontal flip, a 15° rotation, a 30° rotation, and a 15% zoom. Data augmentation was implemented to obtain more samples and to train the hybrid DL model, since the ANNs require a large amount of data to function correctly. In this step, the sequences of images are adjusted to obtain an input tensor of 224 × 224 × 20 per patient. Figure 4 shows an example of the segmentation and data augmentation process for thermal images.

### 2.6. Hybrid Deep Learning Model (CNN-RNN)

#### 2.6.1. Convolutional Neural Network

CNNs have been studied to improve the performance of image classification, image recognition, object detection, and other tasks [46]. CNNs are most used for visual image classification as they allow to extract the information from extensive data, such as images with pixels [47]. Currently, there are different imaging modalities for breast cancer diagnosis, such as mammography, ultrasound, and MRI, and the evaluation of these images is mainly performed with deep learning models such as CNNs [48].

**Figure 5:**
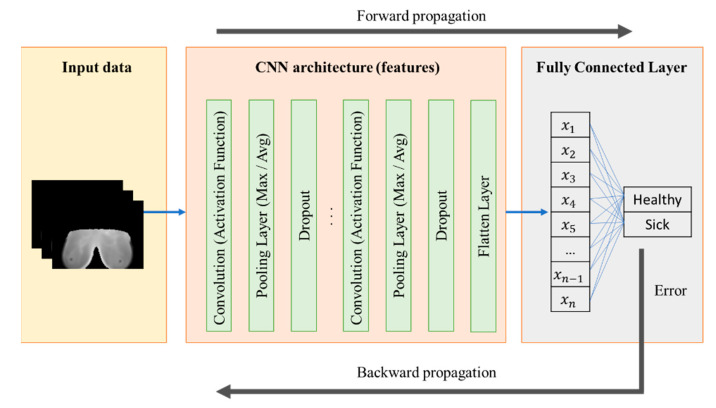
CNN model for the binary classification of breast tissue heterogeneity (normal or abnormal) in thermographic images.

The CNNs are composed of three main layers: the convolutional layer, the pooling layer, and the fully connected layer. The convolutional layer consists of feature learning. Once inputs are in the network, they are used to extract local characteristics from the image at different positions. These convolutions are computed with a kernel to extract several features depending on the values of these small matrices. The results of these convolutions are passed into a nonlinear activation function, i.e., sigmoid, rectified linear unit (ReLU), tanh, or softplus, to obtain a continuous signal [49]. The activation function is an essential part of neural networks as it allows the output to be nonlinear and continuous, enabling the training of the model for either classification or logistic regression [50]. The next main computation is the pooling layer; it extracts features by reducing the dimensions of the feature maps. The most common pooling operations are the average and the max pooling. Finally, the fully connected layer connects all the previous values of the feature vector to apply linear transformations to obtain the product after an activation function. For the classification, the SoftMax regression is the most used in multiclass probability distribution. There is also a procedure known as dropout. It consists of inhibiting a certain number of neurons to retrain the network and ensure robust training. The process of feeding an input into the neural network to obtain the probability distribution in the output layer is known as forward propagation. When there is an error in the regression, this value is considered to retrain the layer in the CNN architecture through the back propagation algorithm. Figure 5 shows the implementation of classifying tissue heterogeneity using CNN architectures. In this figure, it is possible to visualize all the layers and the fully connected layers to obtain a binary classification in the thermal images for the BC disease.

For image classification, there are five CNN architectures in the state-of-the-art: Inception-V3, VGG-16, ResNet101, GoogLeNet, and AlexNet [51]. These architectures were used for automated feature extraction in the hybrid DL model and for classification in the single DL model:

- Inception-v3: It consists of a network of 48 layers where there are 24 parameters to train. It was developed to improve the performance of the GoogLeNet architecture.
- VGG-16: This network consists of a structure of 16 layers, 13 from convolutional layers and 3 of fully connected layers. It is more accurate than the AlexNet architecture, but the training is slower.
- ResNet101: In this architecture, the number of layers is 101. It works by using the residual blocks to optimize the training of the CNN, resulting in an error of 6.44 in the ImageNet dataset.
- GoogLeNet: This CNN architecture has a total of 22 layers, but the number of parameters and the memory of the network size is small. Its training is stable by the auxiliar classifier.
- AlexNet: It is a simple CNN architecture; it is structured by a total of 8 layers, where 5 of them consist of convolutional layers and 3 of fully connected layers. This model can be trained fast, but the accuracy is low in comparison to other architectures.

Once the features were extracted by the five pre-trained CNN architectures, they were reorganized into a vector that was input into three different recurrent neural networks (RNN). As standalone models, RNNs present a considerable challenge when applied to sequences such as raw thermographic image data. Due to their sequential nature, RNNs often struggle with vanishing or exploding gradients during backpropagation through time, especially when working with long input sequences [52]. This limitation reduces their ability to effectively capture long-term dependencies or learn meaningful patterns over extended sequences. In addition, RNNs are not optimized for handling the high spatial dimensionality and intricate patterns present in raw medical images, which results in suboptimal feature extraction performances. A CNN-RNN combination addresses these concerns by using CNNs to extract robust spatial features from each frame of the image before passing these compact, informative representations to the RNN. This approach mitigates gradient problems and ensures effective modeling of both spatial and temporal variations in thermographic image sequences.

#### 2.6.2. Recurrent Neural Network

RNN is a type of artificial neural network that has been used for the analysis of data in sequence in the time domain [53]. This network has demonstrated an enhancement in image and language processing. RNNs are built through intermediate layers representing hidden states where the activation of the actual step depends on the previous step [54]. RNNs work great with small data in sequence, but they have problems with large amounts of data because of the vanishing gradient. Two proposed models based on the original RNN model resolve the problem of gradient disappearance: the LSTM and GRU. LSTM consists of a 3-gate structure to control the information through the memory cells; these are as follows [55]:

- Input gate: It controls the input at the current time step and updates the hidden state of the previous time step.
- Forget gate: It updates the internal state from the previous time step to the actual time step.
- Output gate: It controls the new information into the hidden vector.

The GRU model is a similar model to LSTM but with fewer parameters to update. It is built with two gates: the update gate, which is like the input gate in the LSTM model, and the reset gate, which updates the internal state from the previous time and controls the new information into the hidden vector. Thus, for this study we consider three types of recurrent neural networks: the simple recurrent neural network (RNN), the long short-term memory (LSTM), and the gated recurrent unit (GRU).

### 2.7. Network Specifications

Several CNN architectures were employed to extract features from the thermographic images, including pre-trained versions of Inception-V3, VGG16, ResNet101, GoogLeNet, and AlexNet. These architectures were pre-trained on the ImageNet dataset and adapted to the thermographic imaging context using transfer learning. Specifically, their convolutional bases were used as feature extractors, with weights frozen during training to leverage their pre-trained capabilities. In order to integrate the thermographic image sequences, the input layer of the combined model was adapted to accept a sequence of 20 frames per sample, each resized to 224 × 224 pixels and normalized to a range of [0, 1]. The CNNs were implemented as part of a TimeDistributed layer, enabling feature extraction from each frame independently before passing the extracted features to the RNN layers. On the output side, the classification task was binary (healthy vs. sick), so the final layer was modified to include a single dense neuron with a softmax activation function for probabilistic binary classification. A sequential training process was used to train the CNN and RNN components of the model. The extracted features from the CNNs were processed by different types of RNNs, including simple RNNs, GRUs, and LSTM networks, to capture temporal dependencies in the thermographic image sequences. Each RNN configuration consisted of two stacked layers, each with 64 units. The first recurrent layer was configured to return sequences, enabling the second layer to process the full temporal information of the thermographic data. For the GRU and LSTM models, the forget and update gate mechanisms allowed for effective learning of long-range dependencies while mitigating the vanishing gradient problem typically encountered in standard RNNs. All RNNs utilized the ReLU activation function for hidden units to improve stability during training. The recurrent networks were trained from scratch, with random weight initialization provided by TensorFlow using the Glorot uniform initialization method to ensure stable gradient propagation. Input sequences consisted of 20 thermographic frames per patient, normalized to a range of [0, 1]. The model was trained using the ADAM optimizer with a softmax activation function in the last layer. The learning rate was set to 0.001 per iteration, with a batch size of 16 samples. The number of epochs was set at 30, which ensured model convergence while avoiding overfitting.

**Figure A1:**
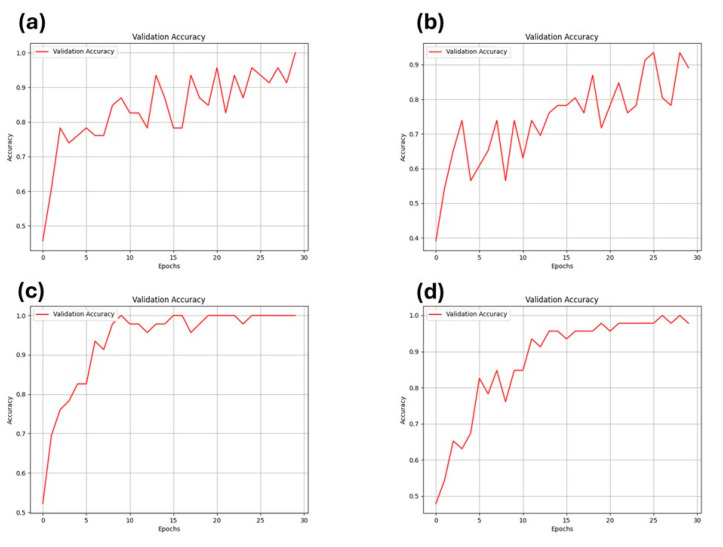
Validation accuracy of InceptionV3 using (**a**) single CNN; (**b**) coupled RNN; (**c**) coupled LSTM; and (**d**) coupled GRU.

**Figure A2:**
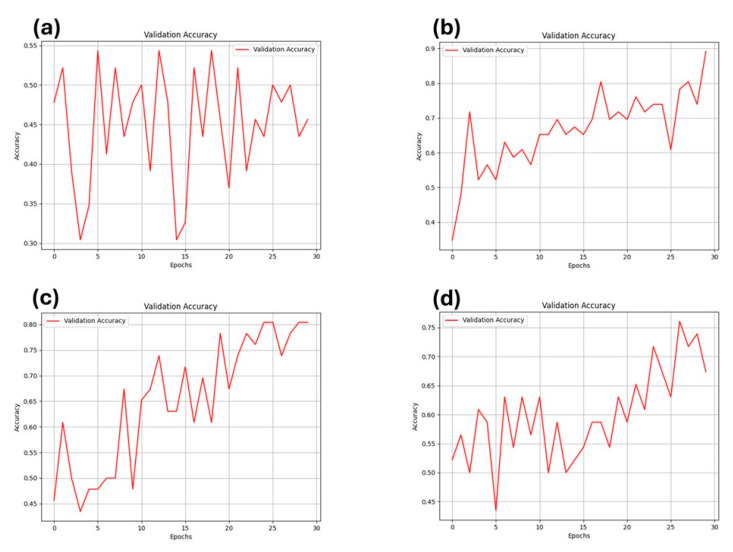
Validation accuracy of VGG16 using (**a**) single CNN; (**b**) coupled RNN; (**c**) coupled LSTM; and (**d**) coupled GRU.

**Figure A3:**
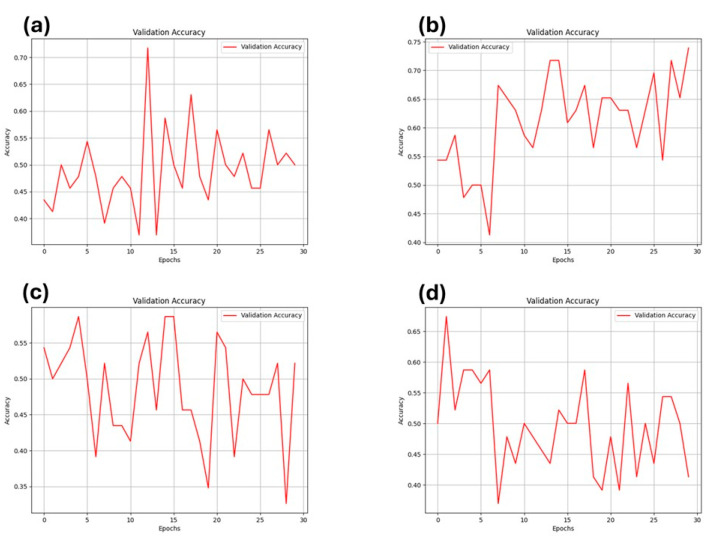
Validation accuracy of ResNet101 using (**a**) single CNN; (**b**) coupled RNN; (**c**) coupled LSTM; and (**d**) coupled GRU.

**Figure A4:**
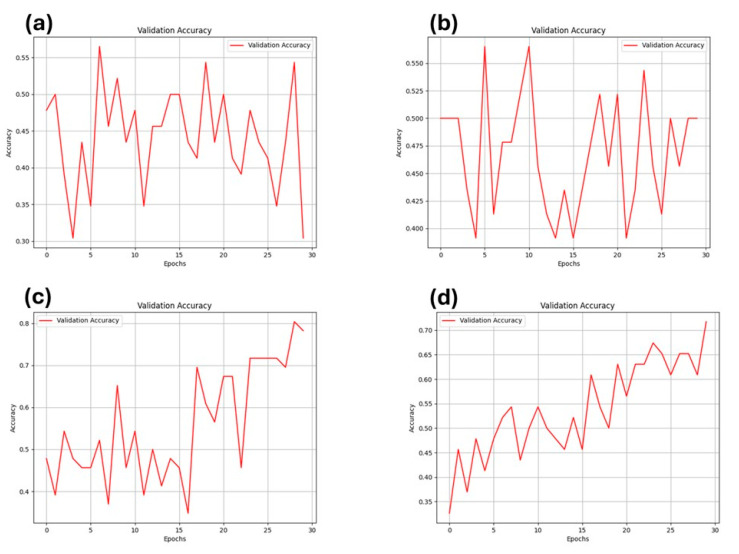
Validation accuracy of AlexNet using (**a**) single CNN; (**b**) coupled RNN; (**c**) coupled LSTM; and (**d**) coupled GRU.

**Figure A5:**
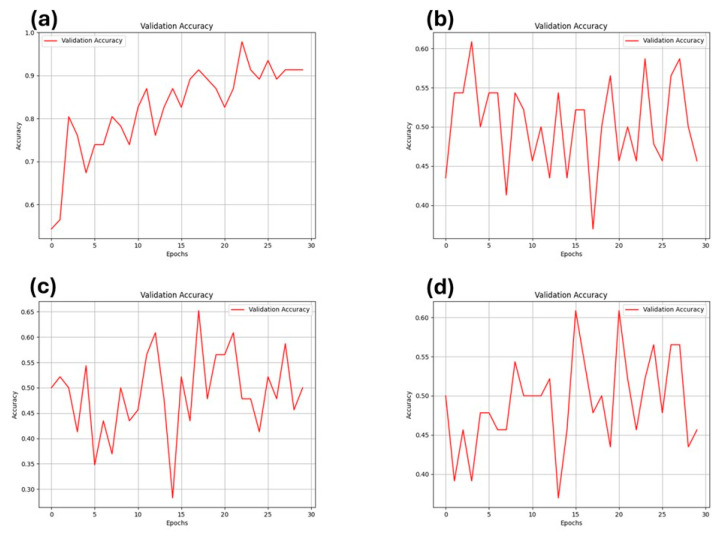
Validation accuracy of GoogLeNet using (**a**) single CNN; (**b**) coupled RNN; (**c**) coupled LSTM; and (**d**) coupled GRU.

To monitor the performance of the models during training, the validation accuracy was evaluated at the end of each epoch. In this way, we were able to track the model’s learning progress and detect overfitting potential. In Appendix A, Figure A1, Figure A2, Figure A3, Figure A4 and Figure A5 illustrate the validation accuracy of the different models.

### 2.8. Performance Metrics

#### 2.8.1. Segmentation Performance

The automatic segmentation of thermographic breast images was validated using the Dice Coefficient and Jaccard Index, two widely used metrics for evaluating segmentation performance [56,57]. The Dice Coefficient measures the similarity between two sets by calculating the overlap between the predicted segmentation and the ground truth, expressed as follows:

(1) $$\mathrm{Dice}=\frac{2|A\cap B|}{\left|A\right|+|B|},$$

where *A* is the predicted segmentation and *B* is the ground truth. Dice coefficient values range from 0 to 1, which denotes no overlap, up to 1, which denotes perfect overlap.

The Jaccard Index, also known as the intersection over union (IoU), quantifies the proportion of overlap between the predicted segmentation and the ground truth relative to their union, calculated as follows:

(2) $$\mathrm{IoU}=\frac{\left|A\cap B\right|}{\left|A\cup B\right|},$$

Like the Dice coefficient, its values range from 0 to 1, where higher values indicate better segmentation accuracy.

The metrics obtained were as follows:

- Average Dice coefficient: 0.9347 ± 0.0138.
- Average Jaccard Index: 0.8776 ± 0.0242.

#### 2.8.2. Classification Performance

The single and hybrid DL models were evaluated to classify the thermal images sequentially from the DTI acquisition approach. For the single DL model, an FCL was set in the last layer of each CNN architecture, unlike the hybrid DL model, where in the last layer of the CNN there are two layers of RNN cells (RNN, LSTM, and GRU). The model was evaluated using the following performance metrics:

(3) $$\mathrm{Accuracy}=\frac{TP+TN}{TP+TN+FP+FN},$$

(4) $$\mathrm{Sensitivity}=\frac{TP}{TP+FN},$$

(5) $$\mathrm{Specificity}=\frac{TN}{TN+FP}$$

where *TP* is the prediction of a sample for the sick class when the real class is sick, *TN* is for a healthy predicted class when the real class is healthy, *FP* is the prediction of a sick class when the real is healthy, and *FN* is for the prediction of a healthy class when the real class is sick. The metrics were computed from the resultant confusion matrix. A CPU execution time was also calculated, which shows the time in seconds required to predict a class using a single and hybrid DL model, considering the input complexity, CNN architecture, and system requirements.

In this methodology, leave-one-out cross-validation (LOOCV) was employed to assess the model’s performance. For each iteration, the model was trained using all data samples except one, which was reserved as the test set. This procedure was repeated for every sample in the dataset, ensuring that each thermal image sequence was used as a test case once. The performance metrics from all iterations were then averaged to obtain a comprehensive evaluation of the model’s generalization capability.

### 2.9. System Requirements

The pipeline was implemented on a machine with the requirements indicated in Table 1, which considers the computer’s specifications and the language and libraries required.

## 3. Results

The CNN architectures—Inception-v3, VGG16, ResNet101, GoogLeNet, and AlexNet—were assessed when coupled with RNN, LSTM, and GRU to classify abnormalities in the breasts with thermal images in sequence. The DL models were evaluated through performance metrics, and the validation used was the LOOCV.

**Figure A6:**
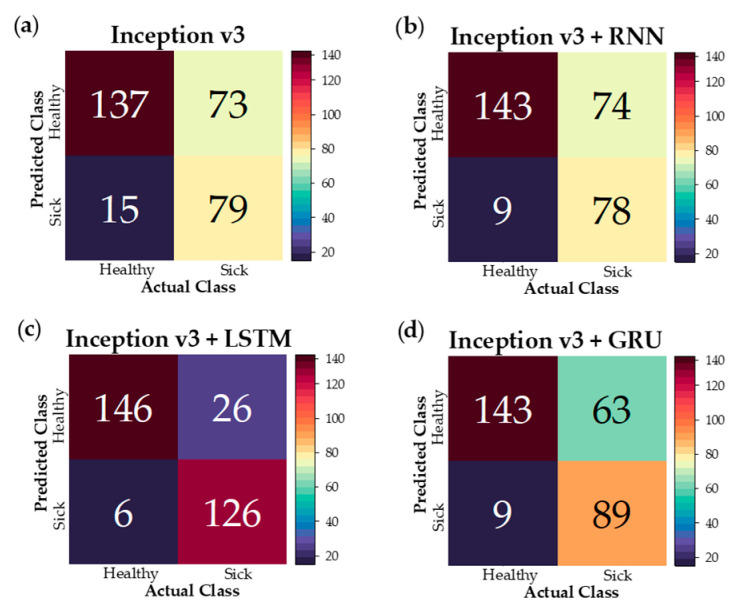
Confusion matrix of InceptionV3 using (**a**) single CNN; (**b**) coupled RNN; (**c**) coupled LSTM; and (**d**) coupled GRU.

**Figure A7:**
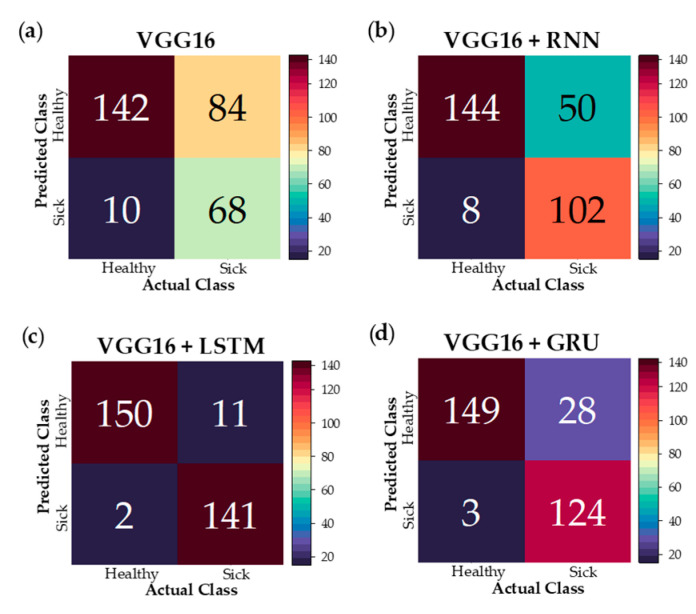
Confusion matrix of VGG16 using (**a**) single CNN; (**b**) coupled RNN; (**c**) coupled LSTM; and (**d**) coupled GRU.

**Figure A8:**
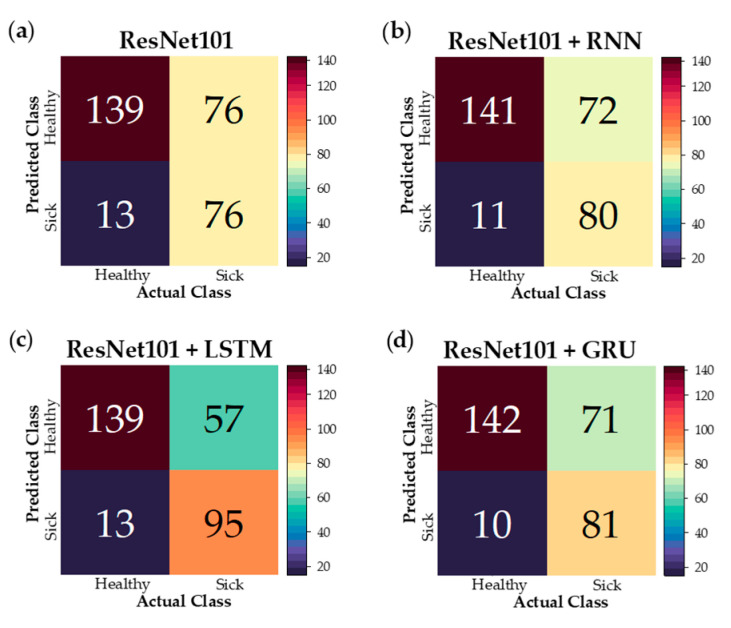
Confusion matrix of ResNet101 using (**a**) single CNN; (**b**) coupled RNN; (**c**) coupled LSTM; and (**d**) coupled GRU.

**Figure A9:**
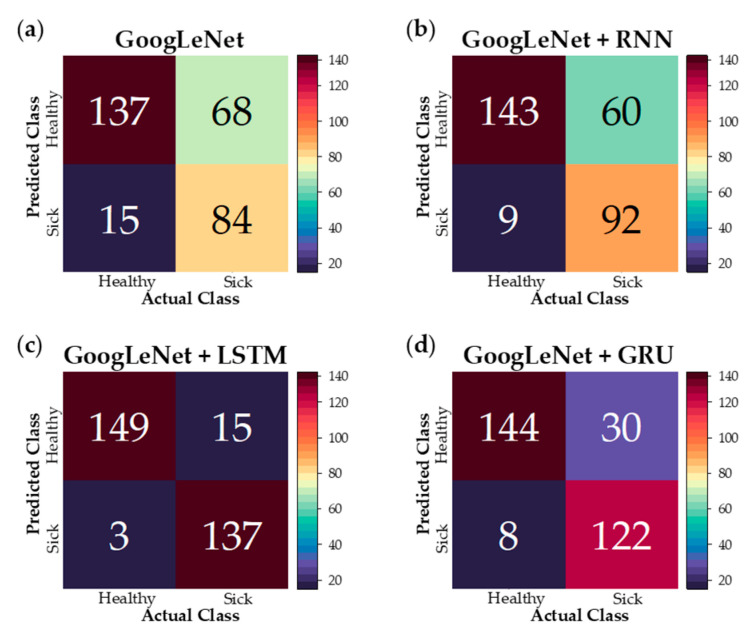
Confusion matrix of GoogLeNet using (**a**) single CNN; (**b**) coupled RNN; (**c**) coupled LSTM; and (**d**) coupled GRU.

**Figure A10:**
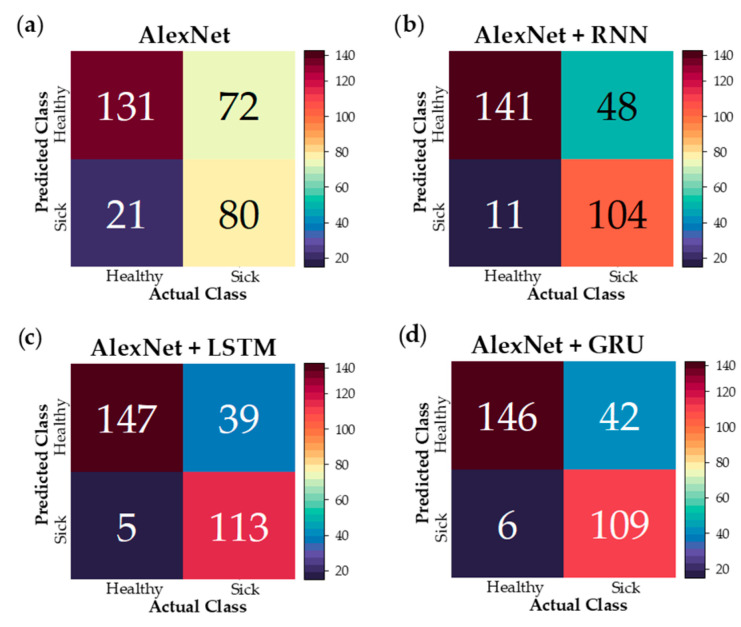
Confusion matrix of AlexNet using (**a**) single CNN; (**b**) coupled RNN; (**c**) coupled LSTM; and (**d**) coupled GRU.

The viability of selecting several datasets to acquire more samples was studied, but none of them included the DIT acquisition approach. In the Visual Lab DMR dataset, the number of sequences obtained was 38 for each class, resulting from the maximum number of samples in the class labelled as sick. Moreover, the data were balanced with a random sampling of 38 sequences from volunteers labelled as the healthy class. Balancing the data was performed to train the model with the same number of samples from each class. In addition, the data was increased with the transformations of data augmentation techniques because of the small number of samples in the training of DL models using a horizontal flip, a 15° rotation, a 30° rotation, and a 15% zoom. Table 2 depicts the number of thermal images in sequence from the healthy and sick classes using filtered and augmented data, and Table 3 shows the performance from the single and hybrid DL models, whose metric values are derived from the confusion matrices of each model (see Appendix B, Figure A6, Figure A7, Figure A8, Figure A9 and Figure A10).

The proposed CNN-RNN binary classifier obtained the highest metrics when VGG16 is used with the LSTM layers, reaching a total of 95.72%, 92.76%, and 98.68% in accuracy, sensitivity, and specificity, respectively (Table 3). On the other hand, the worst performance was achieved from the single DL model AlexNet with 69.41%, 52.63%, and 86.18% in accuracy, sensitivity, and specificity, respectively. According to Table 3, the architecture with the fastest CPU execution time is the AlexNet, a single CNN, with a time of 0.45 s. However, its performance metrics are below those of other models, with accuracy, sensitivity, and specificity of 69.41%, 52.63%, and 86.18%, respectively. On the other hand, the model that obtained the best performance metrics (VGG16-LSTM) takes almost nine times longer in CPU execution time than the single AlexNet model.

Additionally, there is evidence that any of the CNN architectures used in this work in a hybrid DL model (LSTM-based) increases the performance metrics in comparison to the single DL model or the remaining RNN cells, not to mention that the hybrid DL models tested acquired better performance than the single DL model (see Table 4).

**Figure 6:**
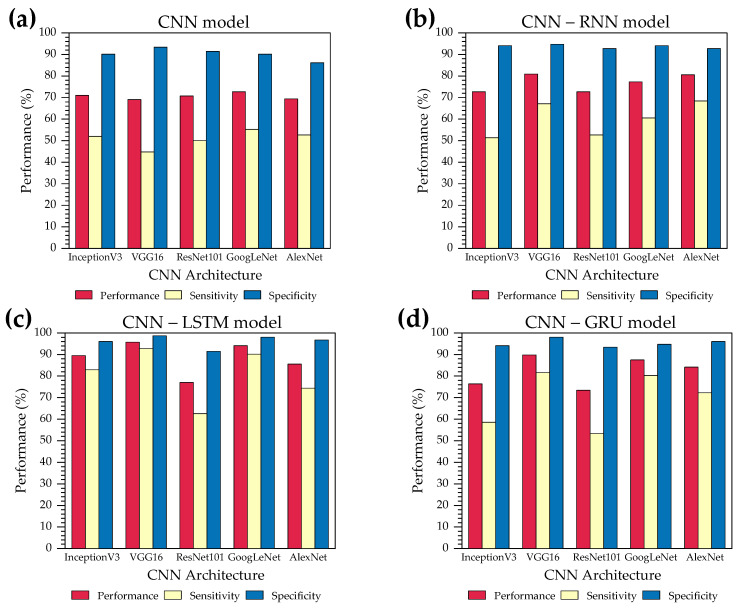
Performance evaluation (accuracy, sensitivity, and specificity) of the different hybrid CNN-RNN architectures to classify the presence or absence of a tumor in breast thermographic images: (**a**) The independent CNN model; (**b**) The hybrid CNN-RNN model; (**c**) The hybrid CNN-LSTM model; (**d**) The hybrid CNN-GRU model. Inception-V3, VGG16, ResNet101, GoogLeNet, and AlexNet are the five CNN models that are coupled to the three sequential networks (RNN, LSTM, and GRU).

**Figure 7:**
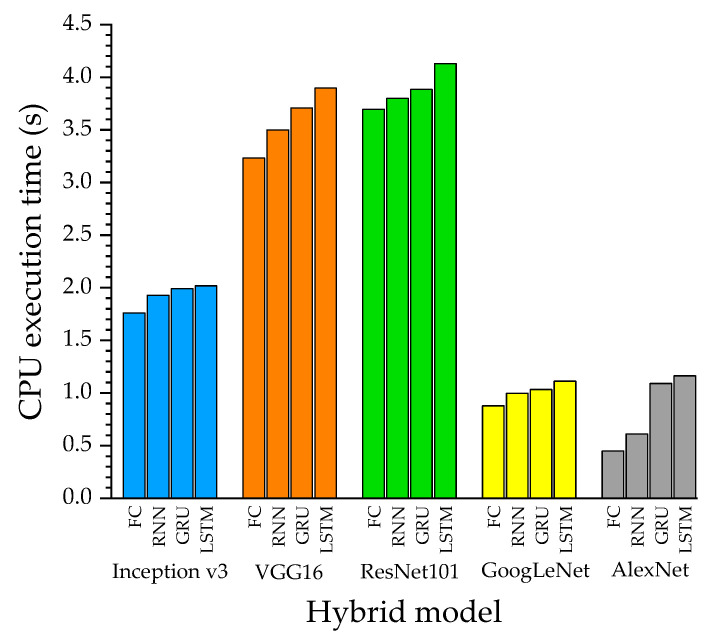
CPU execution time of different coupled CNN-RNN deep learning architectures for breast cancer classification in images acquired using the DIT acquisition protocol.

Figure 6 presents a visual representation of the performance metrics with the different DL models. Here, we compare the results from various CNN architectures with their respective RNN cells or the FCL. Figure 7 shows a comparison of the CPU execution time in seconds of the different models evaluated, either with the deep learning model alone or coupled to an RNN.

The performance metrics calculated by the single CNN models, as well as by CNN models coupled with RNN, LSTM, and GRU, were augmented using the bootstrap method. This approach allowed for the calculation of confidence intervals for each class. The results showed a confidence interval of 69.08 to 71.5 for the simple CNN model, while for the coupled models, the intervals were 72.7 to 80.92, 85.53 to 95.72, and 76.32 to 89.8 for CNN with RNN, LSTM, and GRU, respectively. An ANOVA (analysis of variance) was performed to assess whether there were significant differences between the performance metrics of the models. ANOVA is a statistical method used to compare the means of three or more groups to determine if at least one group differs significantly from the others. In this case, the *p*-value was less than 0.05. This indicates that there is a highly significant difference between the models, as the *p*-value is far below the common significance threshold of 0.05. Therefore, we can confidently reject the null hypothesis and conclude that the models have statistically different performances.

## 4. Discussion

In this study, breast tissue thermographic image sequences were assessed using a hybrid DL model to identify abnormalities that may indicate BC disease. The hybrid model incorporates a CNN to extract spatial features, a RNN to extract temporal features, and a fully connected layer to determine whether the samples belong to a healthy or sick patient. Few studies for breast cancer disease classification with dynamic acquisition protocol in thermal imaging with machine learning and deep learning models [11,20,58,59,60], have shown a lower false negative rate than SIT. In the last decade, neural networks have attracted much attention from researchers due to the increase in computational capabilities and their application in the detection of complex patterns automatically, as in the case of thermal imaging [12]. For instance, Ekici and Jawzal [61] developed software to extract breast features based on bio-data, image analysis, and image statistics. A CNN model optimized using the Bayes algorithm was used to classify the features, resulting in an accuracy of 98.95%. However, this metric is not adequate since they worked with an unbalanced database, and the CNN architecture does not provide reproducibility information. In the study by Cabıoğlu and Oğul [61], it was shown that by performing transfer learning on the AlexNet architecture, the accuracy for classifying breast thermal images can increase from 89.5% to 94.3% if the database is balanced. However, there was no segmentation process in the images, which increases noise caused by non-interest regions [22]. Although CNNs have gained prominence due to their ability to extract features through pixel-based pattern recognition [22], RNNs perform more effectively when images are sequenced (over time-captured images) [22], making them ideal for temporal feature extraction from images acquired by DIT.

Several studies have reported the use of coupled CNN + RNN networks for the classification of breast cancer disease in different imaging modalities. Wang et al. [34] assessed breast histological images using a CNN + GRU model and obtained an accuracy of 86.21%, while a single DL model achieved an accuracy of 80%. A later study conducted by Srikantamurthy et al. [35] reported that for binary classification of histopathology images of BC, the single DL model had an accuracy of 98.6%, while the hybrid DL model of CNN-RNN reached an accuracy of 99.75%. Likewise, Atrey et al. [37] applied hybrid models (CNN + LSTM) to dual-modality mammography and ultrasound images to improve early detection of breast cancer, leading to an increase in classification accuracy from 88.73% to 99.35%.

In this context, this study evaluated the efficiency of coupled deep learning models based on convolutional and recurrent neural networks for classifying breast cancer disease in thermal images obtained by the DIT acquisition protocol. Our findings indicate that coupled models can improve the accuracy of dynamic breast thermographic images, since the accuracy of the stand-alone CNN model (single CNN) was 70.56%, while CNN + RNN, CNN + GRU, and CNN + LSTM were 76.84%, 82.23%, and 88.56%, respectively (see Table 4). The LSTM model performed best when coupled with a pretrained CNN model, which corresponds to a hybrid VGG16-LSTM architecture. However, it has been reported that this type of sequential network is computationally expensive when compared to RNNs or GRUs [35]. Therefore, we have addressed not only the performance metrics for classification but also the CPU execution time associated with binary classification to compare the different combinations of coupled models between the pre-trained CNN architectures and the three sequential architectures (RNN, LSTM, and GRU) (see Figure 6). Thus, the hybrid VGG16-LSTM architecture, which developed the best performance metrics (Acc = 95.72%, Sens = 92.76%, Spec = 98.68%), showed a CPU execution time of 3.89 s, making it the second hybrid architecture that required the most CPU time to complete the classification process (the ResNet101-LSTM model took 4.13 s) (see Table 3). This result is due to the higher number of parameters and layers in VGG16 and ResNet101, unlike Inception-v3, AlexNet, and GoogLeNet [35]. These results are consistent with models that took less time, such as AlexNet, which had a CPU execution time of 0.44 s. However, the classification statistics of this stand-alone CNN model are lower (ACC: 69.41%, SENS: 52.63%, SPEC: 86.18%) than other models (see Table 3). This same pre-trained CNN architecture coupled with the LSTM network improved the classification performance (ACC: 85.53%, SENS: 74.34%, SPEC: 96.71%) as well as CPU execution time (1.16 s). Additionally, the stand-alone CNN architecture, known as GoogLeNet (ACC: 72.70%, SENS: 55.26%, SPEC: 90.13%), also demonstrated high classification performance when combined with the sequential LSTM neural network (ACC: 94.08%, SENS: 90.13%, SPEC: 98.03%), requiring only 0.15 s of CPU execution time over the single CNN model ( GoogLeNet). This time that is negligible when compared to the increase in binary classification performance metrics for determining whether a breast thermographic image contains a tumor.

A limitation of this study is the relatively small dataset, which comprises only 38 sequences per class after balancing. While data augmentation is a commonly used approach to expand sample size and reduce overfitting [39], medical images present a unique challenge due to their inherent complexity and variability. As a result of these characteristics, more advanced techniques are required to ensure that the model can effectively capture the specific features and variability of medical conditions. One possible solution is to use deep generative models, such as VAEs, GANs, and DMs, which have shown promise in generating realistic, diverse images that can improve training by better representing the underlying distribution of the dataset [62].

In the present study, however, the limitation of the small data set persists, since our approach involves analyzing sequential thermography images, and the only dataset with such images (DTI) is the DMR dataset from Visual Lab. In view of restricted access to patient data and the complexity of collecting thermal imaging data, we were not able to create a larger dataset. Thus, the current model is not suitable for widespread clinical application. Nevertheless, with further data collection, this model may contribute to the early detection of breast cancer by aiding clinicians in identifying areas of concern in thermal images, along with other diagnostic tools. The integration of this model into existing clinical workflows is also a critical issue. In spite of the fact that our model has not yet been applied in clinical settings, we consider it to be a potential supplementary tool for radiologists and clinicians. It may be possible to provide additional insights into breast cancer diagnosis by analyzing thermal images alongside other diagnostic methods. However, it would be necessary to address a number of issues to make the model suitable for clinical use, including the processing of real-time data, the design of user interfaces, and the compatibility with existing medical technology.

## 5. Conclusions

Deep learning plays an important role in detecting complex patterns in medical images, making them more reliable, accurate, and faster for diagnosing diseases. In this study, we address the challenge of analyzing sequential thermal images of the breast using hybrid deep learning models. Unlike static protocols, which capture steady-state images at the same time, our approach benefits from additional information obtained over time through dynamic acquisition. A comprehensive evaluation of stand-alone and coupled deep learning models using pre-trained CNN architectures and RNN cells to classify sequential thermal breast images revealed that the best architecture for classification was VGG16 + LSTM. However, other coupled models, such as GoogLeNet and AlexNet with LSTM, achieved higher classification accuracy with a shorter CPU execution time than VGG16 with a higher accuracy. The findings suggest that coupled CNN-RNN deep learning models improve classification performance in thermographic breast images obtained by dynamic acquisition protocol without significantly affecting the execution time to distinguish normal or abnormal breast tissue, making it a promising option for preventative breast cancer diagnosis with a considerable time to obtain its result. This suggests that hybrid deep learning models may be implemented in dynamic breast thermography so that spatial (with CNN models) and temporal (with sequential models) features can be extracted for subsequent radiological determination to determine whether tumor tissue exists or is absent. It would be interesting to investigate optimizing features extracted from thermal images in sequence to reduce the computational cost since neural networks require systems to support model computations, particularly when training.

## Figures and Tables

**Table 1:** System requirements.

RAM	8 GB
CPU	1.60 GHz processor, Core-i5, 8th Gen
GPU	Nvidia, 1050
Languages	Version 3.8 Python
OS	64-bit Windows
Libraries	Numpy, Pandas, OpenCV, Scikitlearn, Tensor Flow

**Table 2:** Number of thermal images acquired after application of the filters and transformations.

	Healthy	Sick
Data Cleansing	38	38
Data Augmentation	152	152

**Table 3:** Performance metrics and CPU execution time of the evaluated CNN architectures coupled with RNNs or classifying with the fully connected layer.

Model		Accuracy	Sensitivity	Specificity	CPU Execution Time (s)
Inception v3	FC	71.05%	51.97%	90.13%	1.76
	RNN	72.70%	51.32%	94.08%	1.93
	LSTM	89.47%	82.89%	96.05%	2.02
	GRU	76.32%	58.55%	94.08%	1.99
VGG16	FC	69.08%	44.74%	93.42%	3.23
	RNN	80.92%	67.11%	94.74%	3.49
	LSTM	95.72%	92.76%	98.68%	3.89
	GRU	89.80%	81.58%	98.03%	3.71
ResNet101	FC	70.72%	50.00%	91.45%	3.69
	RNN	72.70%	52.63%	92.76%	3.79
	LSTM	76.97%	62.50%	91.45%	4.13
	GRU	73.36%	53.29%	93.42%	3.88
GoogLeNet	FC	72.70%	55.26%	90.13%	0.88
	RNN	77.30%	60.53%	94.08%	0.99
	LSTM	94.08%	90.13%	98.03%	1.11
	GRU	87.50%	80.26%	94.74%	1.03
AlexNet	FC	69.41%	52.63%	86.18%	0.44
	RNN	80.59%	68.42%	92.76%	0.61
	LSTM	85.53%	74.34%	96.71%	1.16
	GRU	84.16%	72.19%	96.05%	1.09

**Table 4:** Performance metrics of the model coupled after the CNN architectures. Each value represents the mean of each model from Table 3.

Model After CNN Architecture	Accuracy	Sensitivity	Specificity
Fully Connected	70.59 ± 0.08%	50.92 ± 0.62%	90.26 ± 0.28%
RNN	76.84 ± 0.65%	60.00 ± 2.51%	93.68 ± 0.03%
LSTM	88.36 ± 2.26%	80.53 ± 6.11%	96.18 ± 0.32%
GRU	82.23 ± 2.03%	69.17 ± 6.51%	95.26 ± 0.13%

## Data Availability

No new data were created or analyzed in this study. Data sharing is not applicable to this article.

## Appendix A

### Validation Accuracy

Comparison of validation accuracy over epochs for various combinations of CNNs and RNNs. The architectures include Inception-V3, VGG16, ResNet101, GoogLeNet, and AlexNet, each coupled with RNN, LSTM, and GRU layers. The graphs illustrate the performance of each model in capturing both spatial and temporal patterns from breast cancer thermographic image sequences, highlighting the differences in convergence rates and overall accuracy.

## Appendix B

### Confusion Matrix

Confusion matrices illustrating the performance of various CNN-RNN architectures on the classification of breast cancer thermographic image sequences. The models include combinations of Inception-V3, VGG16, ResNet101, GoogLeNet, and AlexNet with RNN, LSTM, and GRU layers. Each matrix provides a detailed breakdown of true positive, true negative, false positive, and false negative predictions, showcasing the ability of each architecture to correctly classify healthy and diseased cases.
